# Evolutionary Comparative Analyses of DNA-Editing Enzymes of the Immune System: From 5-Dimensional Description of Protein Structures to Immunological Insights and Applications to Protein Engineering

**DOI:** 10.3389/fimmu.2021.642343

**Published:** 2021-05-31

**Authors:** Atefeh Ghorbani, Emma M. Quinlan, Mani Larijani

**Affiliations:** ^1^ Program in Immunology and Infectious Diseases, Department of Biomedical Sciences, Faculty of Medicine, Memorial University of Newfoundland, St. John’s, NL, Canada; ^2^ Department of Molecular Biology and Biochemistry, Faculty of Science, Simon Fraser University, Burnaby, BC, Canada

**Keywords:** DNA-editing enzyme, immune response, cancer, gene mutations, cytidine deaminase, AID/APOBEC and ADAR deaminases, protein structure/folding, evolutionary immunology

## Abstract

The immune system is unique among all biological sub-systems in its usage of DNA-editing enzymes to introduce targeted gene mutations and double-strand DNA breaks to diversify antigen receptor genes and combat viral infections. These processes, initiated by specific DNA-editing enzymes, often result in mistargeted induction of genome lesions that initiate and drive cancers. Like other molecules involved in human health and disease, the DNA-editing enzymes of the immune system have been intensively studied in humans and mice, with little attention paid (< 1% of published studies) to the same enzymes in evolutionarily distant species. Here, we present a systematic review of the literature on the characterization of one such DNA-editing enzyme, activation-induced cytidine deaminase (AID), from an evolutionary comparative perspective. The central thesis of this review is that although the evolutionary comparative approach represents a minuscule fraction of published works on this and other DNA-editing enzymes, this approach has made significant impacts across the fields of structural biology, immunology, and cancer research. Using AID as an example, we highlight the value of the evolutionary comparative approach in discoveries already made, and in the context of emerging directions in immunology and protein engineering. We introduce the concept of 5-dimensional (5D) description of protein structures, a more nuanced view of a structure that is made possible by evolutionary comparative studies. In this higher dimensional view of a protein’s structure, the classical 3-dimensional (3D) structure is integrated in the context of real-time conformations and evolutionary time shifts (4^th^ dimension) and the relevance of these dynamics to its biological function (5^th^ dimension).

## Introduction

The adaptive immune system in its classical mammalian form first appeared in the common ancestor of all jawed vertebrates (gnathostomes), with the cartilaginous fish being the first extant animals to evolve somatically diversified lymphocyte (B and T cell) receptors (BCR or antibodies, and TCR, respectively) (1). However, further study of the earlier-evolved jawless vertebrates revealed that these animals too were capable of adaptive immunity. Instead of B and T cell lymphocytes, their respective humoral and cellular adaptive immune responses are mediated by lymphocyte-like cells with Variable Lymphocyte Receptors (VLRs). Interestingly, these VLRs also appeared to be somatically diversified, highlighting the importance of lymphocyte receptor diversification in the adaptive immune response (2).

Lymphocyte receptors are diversified *via* purposeful induction of DNA damage in the form of recombination and gene mutation (3). Unlike other genes, in jawed vertebrates, the genes encoding the adaptive immune antigen receptors are segmented. To encode a functional receptor, the variable (V), diversity (D; only in the case of the heavy chain), and joining (J) fragments are assembled by V(D)J recombination, a site-specific recombination process that is lymphocyte-specific and mediated by the recombination-activating gene products 1 and 2 (RAG1/2) co-enzyme complex (4–7). Following binding to recombination signal sequences (RSS) at the ends of V, D, or J gene segments, the RAG1/2 complex introduces double strand breaks (DSBs) at the RSS-coding juncture. Non-homologous end joining (NHEJ) is initiated to repair the DSBs, resulting in ligation and forming the V(D)J-encoding gene.

This primary diversification process that occurs during B and T cell development in the bone marrow and thymus respectively, gives rise to the initial antibody (BCR) or TCR repertoire in B and T lymphocytes. In the case of B lymphocytes, further secondary diversification rounds of the BCR are initiated when a mature peripheral B lymphocytes bind its cognate antigen (8). As a result of secondary diversification, activated B cells, expressing low affinity IgM, give rise to B cells secreting high affinity antibodies of switched isotopes including IgA, IgG and IgE. Secondary antibody diversification in jawed vertebrates includes two processes: affinity maturation (AM) and isotype switching (IS), driven by somatic hypermutation (SHM) and Class Switch Recombination (CSR), respectively. SHM in the antibody V region genes, followed by cellular selection leads to antibodies of higher affinity to the cognate antigen. CSR changes the class of antibody from IgM into other isotypes (i.e., IgA, IgG, or IgE). CSR is mediated by DSBs in the switch (S) regions flanking the heavy chain constant genes (C_H_) which initiate a NHEJ event resulting in the replacement of C_H_μ with other C_H_ isotypes, changing the antibody’s effector function (9–11). The outcome of secondary antibody diversification is the production of more effective isotypes of antibodies which also have as much as 1000-fold higher affinity for the antigen. The mutations and DSBs that underlie SHM and CSR are both caused by the enzyme activation-induced cytidine deaminase (AID) (12, 13). AID is a member of the AID/APOBEC (apolipoprotein B mRNA editing enzyme, catalytic polypeptide-like) family of cytidine deaminase enzymes that carry out cytidine (dC) to uridine (dU) conversion in single stranded DNA or cytidine (C) to uridine (U) conversion in RNA (14, 15).

**Figure 1:**
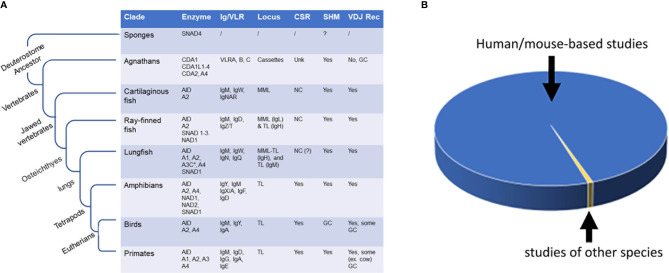
Evolutionary and evolutionary comparative studies of AID/APOBEC and AID/APOBEC-like enzymes. **(A)** The emergence of AID/APOBEC and AID/APOBEC-like enzymes during evolution and the evolution of antibody genes (Ig/VLR), and occurrence of secondary antibody diversification (i.e., antibody maturation; SHM and CSR) and primary antibody diversification (i.e., V[D]J recombination) within and outside of vertebrate class. **(B)** Comparison of number of reports examining AID in human and mouse (blue; > 99%) and studies done on other species (yellow; < 0.7%). The total number of published peer-reviewed studies on AID as measured by AID being a keyword in title/abstract (pubmed/scopus search) is 2368. Of these, 49 discussed the topic of AID in species other than human or mice (15–63) and 17 (0.7%) presented experimental data examining evolutionary divergent AID orthologs (17, 20, 21 26, 30–33, 39, 42, 45, 47, 52, 56, 61–63). NC, noncanonical; MML, multiple mini loci; TL, translocon; GC, Gene conversion (or Gene conversion-like); unk, unknown.

The AID/APOBEC family includes 11 family members in humans: AID, APOBEC1, APOBEC2, APOBEC3 (A-H, excluding E), and APOBEC4. APOBEC4 and related enzymes have been found as early as Cnidarian invertebrates but are frequently absent in actinopterygians and present again in all mammals (14, 16) (**Figure 1A**). The APOBEC3 sub-branch emerged in mammals followed by rapid expansion and diversification in primates (16, 64) (**Figure 1A**). APOBEC3s function in immune response by acting as restriction factors against viruses. They do so through mediating mutagenesis of viral genomes, or interference with the reverse transcription and integration of the viral DNA (65–68). In addition the adenosine deaminases acting on double-stranded RNA (ADARs) are enzymes that mediate cellular mRNA processing through Adenosine (A) to Inosine (I) conversion; however, they have also been demonstrated to mutate viral RNA and carry out a range of cytoplasmic innate anti-viral functions (69–73).

In contrast to jawed vertebrates, the jawless vertebrate lamprey lacks a classical antibody and TCR, and their antibody structure is grossly different, both at the genetic and protein levels. Rather than the classical V(D)J recombination-based Ig system of jawed vertebrates, lampreys employ a presumed gene conversion-like process to assemble 8-10 variable leucine rich repeat motifs in between conserved genes that encode N- and C- terminal ends of their antibody protein. Although the jawless vertebrates lack the classical RAG and AID/APOBEC enzymes, the proposed lymphocytes antigen receptor diversification process is thought to be mediated by AID/APOBEC-like cytidine deaminase enzymes denoted CDA (cytosine deaminase) of which there are two sub-types, CDA1 and CDA2, the former group appearing to have multiple enzyme members (2, 17, 74–78).

Though essential for immunity, the DNA-editing enzymes used to diversify antigen receptors also mediate significant off-target genome damage. There are several mechanisms in place to ensure targeting of RAG1/2 to the *Ig* and T cell receptor (*TCR)* genes. These mechanisms include precursor lymphocyte-restricted RAG expression, *CTCF*-binding elements flanking paired RSS sequences, active chromatin markers, active transcription, and stalled RNA polymerase II (79–82). Despite these regulatory mechanisms, RAG is known to cause chromosomal translocation, deletion, and insertions leading to different types of T cell and B cell lymphoid malignancies, and many of these off-target RAG cleavage events are believed to occur through recognition of RSS-like sequences at non-*Ig* loci, termed cryptic RSSs (83, 84). It has also been shown that the excised signal circle can play the role of RSS and cause RAG-mediated DSBs at a cryptic RSSs in a process termed “cut-and-run” (85). RAG-mediated chromosomal translocations, presumably as a form of mis-targeting of V(D)J recombination are implicated in the etiology of chronic myeloid leukemia (CML), leukemias and lymphomas (79, 86, 87).

Mis-targeted activity of AID also causes genome instability and mutations in B cells (88). For example, in patients with chronic myeloid leukemia (CML), AID-mediated hypermutation of tumor repressor and DNA repair genes have been associated with progression into B lymphoid blast crisis and Imatinib-resistance phenotype (89). In diffuse large B cell lymphomas (DLBCL), somatic hypermutation off-targeting has been reported in proto-oncogenes (90). The IGH/MYC translocation that is signature of Burkitt lymphoma (BL) has a frequency that is correlated with AID activity level (91). AID-induced hypermutations have also been observed in chronic lymphoid leukemia (CLL) (92). There has also been evidence of AID-mediated carcinogenesis in germinal center (GC) B cells as the result of Epstein-Barr virus (EBV)-induced AID expression (93). Interestingly, under strong inflammatory stimuli, the premature expression of AID during B cell development creates an opportunity for cooperation between RAG and AID to drive the clonal evolution of childhood B cell acute lymphoblastic leukemia (B-ALL) (94). The role of AID in tumorigenesis has been conclusively established in several mouse models. In mouse models of IgH/MYC translocation-driven BL, AID has been shown to be directly responsible for this tumor-driving chromosomal translocation (95), and AID transgenic mice are also prone to AID-driven tumorigenesis (96).

In addition to AID, its APOBEC relatives, the APOBEC3 sub-branch of enzymes (A3A, A3B, A3H), which have antiviral properties, are also a significant source of genome damage and mutations implicated in many types of cancers, such as breast, ovarian, and lung cancers, as the driving mutation and cancer progression associated signatures (68, 97–108). Their mutagenic activity in tumors is often the most prevalent mutational signature, and overall, only second to aging-related mutations signatures. In addition to AID/APOBEC cytidine deaminases, recent evidence also implicates ADARs as sources of mRNA mutations in cancer (109–112). Like AID, the role of APOBEC enzymes in tumor initiation has also recently been established in APOBEC-transgenic mouse models (113).

The diversification of the adaptive immune antigen receptors is the only vertebrate example of controlled self-DNA editing and damage in the form of purposeful mutation and rearrangement. The RAG, AID/APOBEC, and ADAR DNA-editing enzymes play important roles in adaptive and innate immunity through the mutagenesis and recombination of the endogenous *Ig* genes, and the response to viral infection. The importance of these enzymes is underscored by the immunodeficiency disorders caused by their deficiency: severe combined immunodeficiency (SCID) and Hyper IgM syndrome in the case of dysfunctional RAG and AID, respectively (114–121). On the other hand, these enzymes also mediate considerable disease-driving collateral damage to the genome. Given their importance to immunity, infection, and cancer, it is not surprising that the DNA-editing enzymes of the immune system have been the topic of intense study in various fields including immunology, virology, cancer, DNA damage/repair and structural biology. In the next section, we provide an overview of the methodological and model organism landscape of this research area. The central thesis of this review is that the evolutionary aspect of these enzymes, despite being an understudied area, has provided key insights from the basic biological and applied biomedical perspectives.

## Central Thesis: Despite the Overwhelmingly Anthro- and Murine-Centric Approaches to Study DNA-Editing Enzymes, Evolutionary Comparative Studies Focusing on Divergent Species Have Provided Significant Insights

A survey of published literature on PubMed/Scopus reveals ~5000 articles focusing on the DNA-editing and DNA-damaging enzymes of the immune system (RAGs: 729, AID: 2368, APOBECs: 2628, wherein these enzymes are in the title/abstract), published over the last 3 decades of work on RAGs and 1-2 decades of work on AID/APOBEC/ADARs. In the remainder of this article, because our work has mostly focused on AID, we will use this enzyme as a representative example of a genome-editing enzyme that has been extensively studied for 20 years [since its discovery in 1999 – (12, 13)] in the fields of immunity, cancer, DNA damage/repair, and epigenetics. In the following paragraphs, we examine the themes, experimental approaches and model systems used to study AID. The principles discussed and the conclusions reached at the end of this review in the context of AID apply equally and in the same manner to other DNA/RNA-editing enzymes involved in immunity (APOBECs, RAGs, and others, discussed below), and, for that matter, to the study of all other molecules that play roles in human health and disease.

First, in terms of study themes, topics of investigation include: understanding (1) functions, including “normal” immune functions (antibody diversification), non-immune biological functions (epigenetic regulation of the genome), and deleterious functions as a result of mis-expression or mis-targeted activity (initiation and progression of cancers), (2) regulation, including regulation of expression, interacting partners (protein, DNA or RNA), and regulation of the targeting of these enzymes to specific genes or genomic loci, (3) networks of cellular processes including for instance the DNA repair and damage response pathways activated downstream of these enzymes’ mutational activities, (4) molecular mechanisms, including biochemical analyses, and (5) 3D structure determination.

Second, in terms of methodological approaches, studies fall into several categories: (1) whole animal *in vivo*, (2) mechanistic experiments using primary cells *or* model cell line *ex vivo*, (3) genomics or bioinformatics studies examining genome-altering signatures of these enzymes, and association with immunity or cancer, (4) structure determination by crystallography or nucleic magnetic resonance (NMR) or emerging computational methods, (5) “simple” cellular experimental systems such as bacteria or yeast in which the enzyme is exogenously expressed followed by reporter assays, (6) biochemical reductionist cell-free studies of the enzymes as purified molecules, *in vitro*.

Third, in terms of model organisms, which will be the focus of this review, for DNA/RNA-editing enzymes involved in immunity and cancer, and indeed for most molecules that play roles in human health and disease, the vast majority of research has been focused on human and, to a lesser extent, mice. For the past several decades, cellular and molecular biology approaches for studying molecules involved in human health focused almost entirely on a handful of well-characterized model species, including the fruit fly *D. Melanogaster*, the worm *C. elegans*, and rodents, most notably lab mice. There are several reasons for this: first, many disease-related molecules function in similar pathways in humans and these model organisms and their dysfunction in the model species closely mirrors the resulting human condition; second, many of these disease-causing molecular pathways are well understood within the model organisms due to decades of research; and third, the model organisms are easy to grow, observe and manipulate at the cellular and genetic levels. Therefore, the concept of studying a handful of model organisms to glean mechanisms of human disease is logical. Indeed, studying molecular mechanisms of human health/disease-related processes in great depth but in a limited number of model organisms is what has led to an unprecedented pace of generating insights into the molecular basis of human diseases.

The total number of studies with AID as the main, or one of the main topics of study, as of the time of preparing this article, is 2368, of which 49 have discussed the topic of AID in species other than human or mice (15–63). Of these, 14 are literature reviews, and of the remaining 35, only 17 studies have presented primary experimental data wherein activities or functional properties of evolutionary divergent AID orthologs were compared (17, 20, 21, 26, 30–33, 39, 42, 45, 47, 52, 56, 61–63). And, among these 17, only less than a handful of studies had an evolutionary comparison as a main conceptual thrust. Therefore, in terms of effort, this area makes up a minuscule (0.7%) subset of the research devoted to the AID enzyme, with > 99% of studies being restricted to human or mouse AID (**Figure 1B**).

The goal of this review is two-fold: Our first aim is to make the case that despite this underrepresentation of effort, several important discoveries have been contributed by working on evolutionary distant AID orthologs, with implications across the fields of cancer, immunity, and genetics. Using the example of AID, we aim to highlight the concept that despite being a road infrequently taken, the evolutionary comparative approach to molecules involved in human health and disease provides immense value for fundamental biological discovery, with emerging practical applications in therapeutics and biotechnology. Our second aim is to suggest that considering the scale of the evolutionary diversity of species, there is an immense knowledge gap in our understanding of DNA/RNA-editing enzymes from species other than human and mouse. In the sections below, we first review the evolution of AID and related enzymes, followed by a review of the contributions made by examining AID through a species-comparative and evolutionary lens, and the future potential of such avenues of inquiry.

## Evolution of AID in the Context of Related Deaminase Enzymes

The AID/APOBEC family is thought to have originated from tRNA adenosine deaminase (Tad)/adenosine deaminase acting on tRNA (ADAT2), the latter of which forms a heterodimer with ADAT3 to deaminate adenosine (A) to inosine (I) in 34 tRNA. These mutated tRNAs can recognize multiple mRNA codons, as I pairs with U, C, or A in the wobble (3^rd^) position (15, 16). Interestingly, ADAT2 may be able to deaminate cytidine in DNA as well (122) indicating the substrate promiscuity of the AID/APOBEC family may have evolved before the APOBEC family divided into the multiple family members. Other enzymes related to Tad/ADAT2, but not to the AID/APOBEC family, include Tad1p/ADAT1, which deaminates tRNA, adenosine deaminases acting on pre-mRNA (ADARs 1, 2, and 3), which is involved in post-translational modifications of RNA (123–125); and cytosine deaminase, cytidine deaminase, and deoxycytidine monophosphate deaminase (dCMP), members of the pyrimidine salvage pathway which recycles nucleotides (126). These enzymes are found throughout the metazoa phylum (16).

Members of the classical AID/APOBEC family (APOBECs 1, 2, 3, and 4) and their newly discovered sister clades and members are discussed below, in the order in which they likely evolved. It is suggested that the AID/APOBEC family has evolved from the tRNA adenosine deaminases containing the consensus motif (C/H)xEx_n_PCxxC (x is any given amino acid) as their catalytic domain (14, 127). The shift in substrate specificity from adenine to cytidine during the divergence of the AID/APOBEC family from Tad2/TadA deaminases has been attributed to the expansion of the α4-β4 loop (i.e., Loop8) and a conserved tyrosine in this loop. The larger L8 decreases the size of the substrate-binding pocket, and the conserved tyrosine could participate in base-stacking interactions (128). Moreover, the HxEx_n_PCxxC motif is the conserved catalytic domain shared by the AID/APOBEC family in which the glutamate (E) acts as a proton donor and the histidine (H) with two cysteines (C) coordinate a Zn^2+^ ion with the help of a water molecule (39, 52, 129, 130).

The secreted novel AID/APOBEC-like (SNAD) enzymes belong to a sister clade to the classical AID/APOBEC family, evolving in the first animals to diverge from fungi (sponges, SNAD4) and appearing throughout the vertebrate phylum (SNAD1). SNAD2 and 3 found only in the ray-finned fishes are likely the result of whole genome duplication event and/or subsequent expansion of this class. SNAD enzymes are the only AID-like enzymes in multicellular eukaryotes with a characteristic predicted secretion sequence and have therefore been proposed to be secreted potentially for delivery to virus-infected cells or extracellular parasites; however, their catalytic activity and other biochemical characteristics remain unknown. They may have originated from bacterial toxin proteins (16).

APOBEC4 (A4), a member of the classical AID/APOBEC family, was likely next to evolve, first appearing in the cnidarians (corals), which diverged after sponges (16). The lack of introns in the A4 gene indicates it may be the result of early retrotranspositional events. A4 is present in the first vertebrates, the jawless fish (agnathans), the lobe-finned fish (sarcopterygians), and tetrapods, but is lost in sharks and often lost in ray-finned fishes (actinopterygians). It is expressed in human testes, but its biological role and catalytic activity are unknown. Unlike the other members of the AID/APOBEC family which are known to deaminate polynucleotides, critical amino acids required for polynucleotide deamination (SWS and F in the middle of the deaminase motif HXE….PCXXC) are missing from A4, indicating it may act on other substrates (15, 16, 131).

The next-evolved branch of AID-like enzymes include cytidine deaminase-like 1 (CDA1), CDA1L1, 2, 3, and 4, and CDA2 found in the jawless vertebrates (agnathans). Lampreys lack many canonical “pillars” of the adaptive immune system, such as RAGs and MHC; however, they do have antibody-like proteins (VLRs) that are diversified somatically, which led to the discovery of CDA1-like, and CDA1 and 2 in the freshwater and sea lampreys, respectively. These enzymes will be discussed in detail in a following section (16, 17, 20).

This was followed by the emergence of AID and APOBEC2 (A2) in the common ancestor(s) of jawed vertebrate classes of cartilaginous and bony fish. Hence, A2 and AID are considered the ancestral family members of the classical APOBEC family due to being present in most jawed vertebrates tested to date. They appeared at the same evolutionary juncture where the classical V(D)J-based *Ig* recombination and canonical heavy/light-chain based antibody structures emerged (16, 57). Interestingly, the involvement of CDA1 in diversifying the lamprey’s immune receptors and the continuing of a similar role for AID in the jawed vertebrate may be an example of convergent evolution in that the acquisition of the lymphocyte receptor diversification role by the AID-like branch had already occurred before the further divergence of this branch within vertebrates. A2 may be the result of early retrotranspositional events, which used AID as a scaffold. Like A4, human A2 does not appear to edit RNA, DNA, or free cytidine *in vitro*. Its ortholog in zebrafish, which has been implicated in retina and muscle generation, also lacks deaminase activity (132–134). Additionally, A2 seems to inhibit transforming growth factor (TGF)-β in *Xenopus* (frog, amphibian) and zebrafish (135).

The so-called novel AID/APOBEC-like Deaminases 1 and 2 (NAD1/2), while not being original members of the classical AID/APOBEC family, are closer in sequence to A1, A2, A3 than A4. NAD1 is found in ray-finned fishes, the coelacanth (sarcopterygian), amphibians, lizards, and marsupials; NAD2 is found only in amphibians. Neither NAD has been characterized and their biological relevance remains unknown (16).

APOBEC1 (A1) is the founding member of the AID/APOBEC family (136–138). It was originally thought to be first evolved in mammals due to duplication of AID; however, this duplication likely occurred in or before the lungfish. A1 deaminates the cytosine at position 6666 of Apolipoprotein B mRNA, creating a premature stop codon at this position, altering ApoB100 to ApoB48, which is essential for secretion of triglyceride-rich chylomicrons (139). It was later discovered that like AID and A3s (below), A1 is also quite promiscuous, acting on retroviral substrates and ssDNA (140, 141). As A1 is among the later AID/APOBEC family members to evolve, the RNA-editing capabilities seen in other members of this family may be a late-evolved characteristic. On the other hand, due to the progenitors of the AID/APOBEC family acting on RNA and, in some cases, both RNA and DNA (142), substrate promiscuity may be an original characteristic of the many family members, whose activity has just not yet been fully elucidated. In support of this, changes in substrate binding surface regions of the AID and APOBEC-related deaminases appear to be the most rapidly evolving structural feature of these enzymes, and AID certainly appears to recognize RNA and DNA/RNA hybrids with very high affinity though its catalytic activity is restricted to the ssDNA strand (143).

APOBEC3 (A3) is the last group of AID/APOBEC enzymes to have emerged, likely the result of AID’s gene duplication events. A pronounced expansion has occurred most recently in primates leading to 7 unique primate-specific A3 genes (A3A, A3B, A3C, A3DE, A3F, A3G, and A3H) (64, 144). The expansion of these enzymes has been proposed to be due to an arms race between mammals and the targets of A3, retroviruses. The origin of A3 is not fully clear: the initiating duplication event was thought to take place in the first placental mammal where no A3 ortholog were found in animals that diverged before placental mammals. It is thought that in rodents, pigs, and cattle, two AID-like genes fused to form a single gene; in horses, bats, and felines, one of the two genes repeatedly duplicated leading to an expansion of A3 genes. However, the sequenced lungfish genome appeared to contain a putative A3C gene (145). It is possible that the A3C found in the lungfish was a novel APOBEC-like gene representative of convergent evolution.

## The Evolution of Immunoglobulin Loci and Diversification

Pre-vertebrates (protochordates) lack AID but have AID-like enzymes such as the aforementioned SNADs. While also lacking B cell receptors, these animals have immune receptors belonging to the immunoglobulin superfamily (146–149). It is believed that a type of proto-AID (or AID ortholog) was present in the first vertebrate ancestor, which then diverged to CDA in the lamprey and to AID in the early jawed vertebrates, the shark (17, 18). Similarly, it is hypothesized that the targets of this proto-AID (somatically diversified lymphocyte receptors) diverged into three unique receptors with three different lymphocyte cell lineages: a secreted form (VLRB in the lamprey and BCR in jawed vertebrates in B cell-like cells) and two membrane-bound receptors (VLRA/C in the lamprey and TCR αβ/γδ in jawed vertebrates in T cell-like cells) (18, 150). Due to CDA1/1L genes lacking introns, it has been posited that CDA2 was the original enzyme in all three lamprey lineages, with the ability to somatically diversify all three VLRs, and that CDA1/1L genes were the result of retrotransposon events after which CDA2 was subsequentially silenced in CDA1/1L^+^ cell lineages. This idea is supported by the fact that in the first-diverged subsequent jawed vertebrate, the shark, AID appears to initiate somatic hypermutation of both B and T cell receptors (19, 35, 151, 152). This suggests that perhaps this broader dual role of AID was lost in subsequent vertebrate lineages and the role became focused on antibody diversification in B cells only but the dual role appears again in limited later-diverged species, such as the Ballan Rasse (ray-finned fish) and in camels (36, 153). Lamprey CDAs have been relatively understudied after their discovery, with their VLR antibodies garnering the most attention as novel non-classical antibody structures that may hold biotechnological and therapeutic potential (154–156).

The first immunoglobulin loci to evolve were those in the elasmobranchs (sharks and skates) that are organized quite differently from the most-studied mammal *Ig* loci. Shark *Ig* loci are organized into multiple mini loci (MML) (149, 157), with a mini locus or “cluster” equating to one V region placed next to one or more D regions, followed by one J segment and a single constant region (V-DDD-J-C)_n_ (158). Some MML are rearranged in the germline, while most are rearranged by the RAG recombinase. Shark *Ig* undergo SHM, with long, tandem substitutions unique to these species and presumed to be due to AID-initiated mutations (57, 158–162). It was initially believed that shark *Ig* did not undergo CSR; however, though shark Ig sequences lack the conventional switch regions which first appeared in amphibians, recombined VDJ of one cluster can be “switched” with that of another, leading to a different constant region attached to the recombined VDJ region, possibly initiated by AID acting on recombination hotspots in a process that is concomitant with SHM rather than separated as in after the appearance of distinct switch regions (24, 163, 164). The studied Sharks have three types of Ig: IgM, present in almost all vertebrates, IgW (may be a counterpart to IgD), and IgNAR, which is unique to sharks, being made up of only heavy chains (165).

Outside of humans and mice, SHM and CSR have been studied most in ray-finned fish. Poikilotherms such as ray-finned fish have modest changes in antibody affinity, which has been reported in several species to be initiated by SHM (40, 41, 43, 49, 57, 166–168). This is likely due to inefficiencies caused by a lack of organized GCs; instead, ray-finned fish appear to have GC-like clusters of melanomacrophages with AID-producing cells in the centre (41, 169). Teleost fish (ray- and lobe-finned fish) appear to have *Ig* loci made up of both MML and translocon-type organizations, the latter of which is how most tetrapod *Ig* loci are arranged. In ray-finned fish, the V, D, and J segments are arranged as in mammalian *Ig* loci, with the IgM and IgD constant regions at the 3’ end, one after the other. However, the teleost-unique IgZ/T constant region is located further upstream, separated from the IgM and IgD constant regions by D segments (V_n_-D_n_-J_n_-C_Z-_D_n_-C_µ_C_δ_) (57, 157, 170–172). Lungfish also have IgW and the lungfish-specific IgN and IgQ (173). Though bony fish *Ig* loci do not undergo CSR which appears first in amphibians, the IgM and IgD isotopes are “switched” *via* alternative mRNA splicing, while IgZ/T can be expressed after alternative V(D)J rearrangement.

## Comparative Evolutionary Studies of AID in Cell-Based Functional Assays

The most emphasis outside human and mouse AID has been on fish, because of the expected level of divergence in the primary sequence, and unique features found in fish AID’s primary structure compared to the very well conserved mammalian counterparts. Due to evidence of SHM in the early-diverging vertebrate fish lineages as discussed in the above section, it was hypothesized that an AID ortholog could be found in bony fish, and it was indeed found in channel catfish (Ip-AID) (40). This was the first non-human/mouse AID ortholog to be identified followed by detailed work on tissue expression patterns and possible roles in SHM. Shortly thereafter, it was determined that zebrafish also has a *bona fide* AID gene (Dr-AID) and noted that it, along with the predicted AID genes from other ray-finned fish, encodes an additional 9 amino acids (aa) in the cytidine deaminase motif, along with a different N terminal motif compared to tetrapod AIDs (44). In 2004-2006, a series of early studies looked at the functionality of a small number of fish AID alongside *Xenopus* AID using exogenous expression in bacteria or yeast and measuring mutagenic activity in colony formation reversion assays, or expression in murine or human AID-deficient B cells followed by assaying for CSR (31–33, 49). Even though canonical CSR only occurs in tetrapods (37), multiple fish AID orthologs were able to initiate both mutations in *E. coli*, *S. cerevisiae*, and murine cells and CSR when exogenously expressed in AID-deficient B cells, albeit less effectively than mammalian AID (31–33, 49). This suggested that CSR as it occurs in mammals evolved due to the emergence of switch regions within immunoglobulin loci, and not due to adaptations of the different AID orthologs, and that the poikilotherm AID itself is fully capable of mediating CSR. In depth analyses of the regions of human AID required for CSR pointed to the C-terminus raising the possibility that this region of AID may be important in other biological roles prior to the evolutionary emergence of Ig CSR (174). Importantly, these studies also provide strong opposition to the view that CSR mediation by AID requires a specific set of protein co-factors, because early fish AID are presumably not co-evolved with such presumed co-factors required to chaperone AID to switch regions of the *Ig* genes in mouse cells. These findings are in line with later findings that the role of AID in mediating CSR is simple dC mutation and DSB generation, and that likely AID is targeted to these regions through the abundance of ssDNA structures such as R-loops and DNA/RNA hybrids that are inherently favored by AID (143, 175, 176).

In experiments wherein fish AIDs were exogenously expressed in murine AID-deficient B cells, zebrafish AID and mouse AID could mediate equally efficient CSR, with fugu AID and catfish AID being respectively 4- and 7-fold less efficient than these. Nuclear cytoplasmic shuttling of AID has been shown to be a key regulator of its activity and catfish and fugu AIDs appear to have nuclear export and localization domains conserved with other non-mammalian vertebrate domains with expectant results upon their mutation and it was shown that removal of this domain results in accumulation of AID in the nucleus, confirming its functionality. However, generation of hybrid AID with interchanged NES domains demonstrated that the aforementioned difference in their ability to mediate CSR was not due to different NES sequences, suggesting that fish AIDs may have different inherent catalytic robustness (31, 32, 49, 177). In the same set of experiments, the functionality of *Xenopus* AID was also confirmed for the first time.

Another property of fish AID that was examined in these early studies was temperature sensitivity. It was found that incubating the cells in which the fish AID are being expressed at lower temperatures than the typical 37°C (18°C for bacteria, 30°C for yeast, and 26°C for mammalian cell lines), yielded generally more AID activity in the bacterial colony count, yeast-null mutation, and GFP reversion based assays employed in bacteria, yeast, and cell lines, respectively (15, 31, 32, 49). The lamprey CDA1-class deaminases were also shown in bacterial and yeast-based expression assays to be active cytidine deaminases. Another example of a structure:function insight was the example of using zebrafish AID to propose a role for S38 phosphorylation-dependent interaction of AID with replication protein A (RPA) and its role in mediating CSR. Since zebrafish AID lacks this residue but contains D44 which can act as a phosphomimetic residue, it was proposed that S38 phosphorylation dependent Replication protein A (PRA) interaction is essential for CSR, though another study using a zebrafish AID with a D44 mutation found that this residue is not critical for CSR (30, 42, 62, 178); therefore the importance of this axis of S38 phosphorylation-AID-RPA remains uncertain, as the early view that specific cofactors chaperone AID to the *Ig* locus ought to be considered in balance with the various explanations that it may be the process of transcription and its unique features at the *Ig* loci including robust and bidirectional transcription, and unique DNA or RNA secondary structures (e.g. G quadruplexes) are the determinants that recruit AID to the Ig loci to carry out SHM and CSR (179–182).

As the first tetrapods, amphibian (*Xenopus*) antibodies undergo SHM and CSR; however, the switch regions in *Xenopus* are AT-rich compared to GC-rich, which may affect switching efficiency (183, 184). *Xenopus* AID has been shown to demonstrate CSR activity, and is expressed in hematopoietic tissues, hinting at a role in ontogeny (31, 51). Neither *Xenopus* nor other amphibian AIDs have been biochemically characterized. Avian *Ig* loci, at least the ones sequenced (duck, chicken, and ostrich) are unique among the higher vertebrates in that there is a single functional germline locus (V-D_n_-J or V-J) that is recombined *via* V(D)J recombination; further diversification occurs *via* AID-mediated gene recombination (similar to how VLRs are recombined), initiated by avian AID (185–188). Aside from experiments demonstrating that bovine AID can demethylate DNA *via* deaminase activity (61), no other non-human, non-mouse AID has been characterized in the higher vertebrates, and its targets (*Ig*) and activity (SHM and CSR) in many non-human animals remain unstudied.

## Comparative Evolutionary Analyses of AID in Cell-Free Biochemical Assays

Over the last decade, we have pursued a comparative enzymology approach to study the biochemical properties and structure:function aspects of purified AID from divergent orthologs. The initial goal of this effort was to gain insights into the 3D structure of human AID. Given AID/APOBECs’ involvement in immunity and cancer, intense research has been dedicated to solving their 3D structures. Unfortunately, AID/APOBECs proved to be problematic subjects for X-ray or NMR because they are difficult to make in large quantities due to host cell toxicity, and they form extensive non-specific interactions with other molecules making them hard to purify and insoluble. Hence, > 90% of the 40 reported AID/APOBEC structures are of partial or significantly altered versions, quite a few with < 50% identity to the native protein (PDB databank: https://www.rcsb.org/) (53). These alterations were necessary to enable crystal formation for X-ray crystallography or enhance solubility for NMR. AID is a small (only 198 aa) protein but it has by far the most positively charged surface amongst the AID/APOBEC family, which underlies its exceptionally high binding affinity (~nM-range) for its negatively charged ss-DNA substrate (189). Partially because of this, it has not been possible to obtain a native AID crystal or NMR structure despite intense attempts for 20 years since its discovery in 1999.

Based on the initial insights from the cell-based assays that revealed differences in functional efficiencies of orthologous AIDs and the relatively high divergence among mammalian and fish AID, we posited that AID from more distantly evolved species, might have distinct properties and that discovering the basis of their differences would shed light on AID’s inner workings. We began studying AID from key evolutionary points. Fish were of great interest because they are the most evolutionarily divergent species known to have AID, and their AID sequences exhibit the highest degree of primary sequence divergence. Parallel to the evolutionary approach, several partial X-ray or NMR structures of APOBECs were utilized in computational modelling to generate thousands of predicted AID 3D structures. Through this computational modelling and evolutionary approach, hereafter referred to as the “computational-biochemical-evolutionary” method, parts of AID were predicted to have a specific function. A library of different AID versions (mutants, chimeras with exchanged domains, fish orthologs) was generated, purified, and subjected to functional biochemical enzyme assays (e.g., enzyme kinetics, substrate binding, and optimal temperature determination) to verify whether a motif predicted by the modelling indeed mediated the supposed function. The experimental results were cross-referenced with the evolutionary/computational predictions, in order to refine a functional map of AID’s structure, first published in 2015 through this approach (52). This functional map of AID was later confirmed independently by an X-ray crystal of a near-native AID in 2017 containing 20 aa truncations and a handful of residues mutations which altered the surface charge of AID from ~+10 to +3 (52, 129). In the following paragraphs, we review the insights gained through the computational-biochemical-evolutionary method.

In 2012, by comparing the enzymatic activity and predicted structure of Hs-AID with bony fish AIDs (i.e., zebrafish [Dr-AID] and catfish AID [Ip-AID]), we demonstrated that different AID orthologs present diverse biochemical properties, such as catalytic rate and optimal temperature, which are governed by a single amino acid in their C terminus (26). The difference in the optimal temperature mirrored the ambient temperature of each organism. We observed that Dr-AID was several fold more active than Hs-AID while Ip-AID was significantly less active than Hs-AID, in line with the previous observations of its lesser ability to mediate CSR when deployed in an AID-deficient B cell (26). The different catalytic rates amongst AID orthologs may reflect the different evolutionary paths taken by each species’ immune system. The computational modelling of the surface charge and topology, and functional ssDNA binding assays of bony fish and human AIDs, also led to an early picture of AID’s ssDNA binding grooves. The width of this groove is ~ 10 Å. Given the width of ds-DNA helix (~ 20 Å), the identified DNA binding groove on AID explained its substrate specificity for acting only on ssDNA and not dsDNA (190–193). The presence of this DNA binding groove has been confirmed upon crystallization of Hs-AID with ssDNA (129, 194).

In 2013, we demonstrated that zebrafish AID, unlike its human counterpart and several other bony fish AIDs had the unique enzymatic ability to mutate 5-methyl dC (5mC) in addition to regular dC (39). Soon after its discovery, a possible role of AID in genome methylation and epigenetic reprogramming was suggested where AID demethylation activity in the CpG motifs would convert 5mC to deoxythymidine (dT) (195). Supporting evidence came from the fact that the AICDA gene is located in a cluster with other pluripotency genes and is expressed in oocytes and primordial germ cells (196). Soon after this initial report, AID-mediated deamination of 5-mC was reported in induced pluripotent stem (iPS) cells, primordial germ cells, B cells, cancerous cell lines, and bovine and zebrafish embryo (60, 61, 197–201). Regarding the enzymatic activity of AID on 5-mC, initially, it was claimed that Hs-AID has comparable activity on 5-mC as well (196). However, soon after, several reports showed that although Hs-AID can indeed deaminate 5-mC, its activity on this substrate and on other cytidine derivatives with bulky adducts is many folds less than on dC (39, 202–204). This is a key aspect of AID activity since AID-mediated CpG demethylation through a C to T mutation could be a mutagenic process. Given the importance of CpG motifs in gene expression and epigenetics, one would expect to avoid efficient activity of AID on 5-mC. In fact, methylation has been proposed as a protective mechanism against undesirable AID activity (202). We then used our comparative computational approach and reported that unlike Hs-AID, Ip-AID, medaka AID (Ol-AID), and tetraodon AID (Tn-AID), the zebrafish AID exhibits more efficient activity on 5m-C, deaminating it more efficiently than many other orthologs deaminate regular dC and significantly more efficient as normalized to its own activity on dC (39). From a biological standpoint, these results explained why in zebrafish, AID was uniquely involved in embryonic development and its knockdown resulted in genomes with hypermethylated CpG motifs. From an AID structure:function standpoint, modeling predictions of human and zebrafish AID catalytic pockets docked with dC showed that both AIDs are predicted to form catalytic pockets with the classical triad of Zn-coordinating residues (C87, C92, and H56 in human AID) and catalytic glutamic acid (E58 in human AID) that can accommodate a dC residue in orientations that support the 4-stage deamination chemistry common to cytidine and cytosine deaminase. Importantly, the catalytic pocket of zebrafish AID was predicted to have one of its composing loops extended and more flexible as compared to that of human AID, thus providing more space for a 5mC substrate that is bulkier than a dC (204). In this manner, the computational-biochemical-evolutionary method not only solved a biological puzzle about the role of AID in zebrafish, but it also made a key structural biology contribution by providing the first detailed maps of AID’s catalytic pocket through predictive modelling corroborated with functional enzymology.

In 2015, using our computational-biochemical-evolutionary method, we mapped a network of primary and secondary catalytic residues that either contact and/or stabilize the dC in a catalytic pocket (52). This network of amino acids consists of G23, R24, R25, E26, T27, L29, N51, K52, N53, G54, C55, V57, T82, W84, S85, P86, D89, Y114, F115, C116, and E122 in human AID (52). These residues form the “walls” and “floors” of the catalytic pocket and interact with substrate dC in several predicted protein conformations through hydrogen bonding, electrostatic interactions, and aromatic base stacking. The importance of direct interactions between some of the secondary catalytic residues and substrate DNA was validated when the crystal structure of a partially truncated and mutated but relatively near native AID was published (129). Given the importance of proper positioning of dC inside the active site for efficient deamination activity, defining the secondary catalytic pocket residues was a step forward in solving the functional structure of AID. In the same work, we also described a novel structural regulatory mechanism of AID/APOBEC activity in that the majority of Hs-AID conformations at any given time contain catalytic pockets that are closed and inaccessible for accommodating a dC for deamination (194). Furthermore, we observed that the majority of ssDNA:AID binding events result in ssDNA bound non-productively on the surface in conformations that do not pass over the catalytic pocket, presumably due to the highly positively charged surface of AID (+11, the highest surface positive charge amongst AID/APOBECs) (52, 194). Taken together, the frequent catalytic closure and sporadic ssDNA binding are significant bottlenecks for AID activity such that < 1% of all ssDNA:AID binding events translate into a cytidine deamination event. We then proposed that due to the potential danger of AID/APOBEC activity for genomic DNA, this inherent structural regulatory mechanism is in place as a safe-guard mechanism in AID and in the tumorigenic A3 family members; the main pillar of this hypothesis was that the open:closed dynamic ratio in AID, A3A and A3B correlated with their catalytic rates and with their relative responsibility for mediating tumorigenic mutations in cancers. We termed this novel mechanism Schrödinger’s CATalytic Pocket (53). Here again, the computational-biochemical-evolutionary method was key in providing the functional proof for the existence and regulatory role of Schrödinger’s CATalytic Pocket. A panel of chimeric AID enzymes, including bony fish-human chimeras, was generated since certain fish AID (e.g., the aforementioned zebrafish AID) have catalytic pockets are composed of loops of different lengths and hence different breathing dynamics compared to human AID. The demonstration that the AID chimeras (*e.g.*, human/zebrafish catalytic pocket chimera AID, or AID/A3 chimera) predicted to spend more time in the open conformation also have higher catalytic rates, provided functional proof of the concept for Schrödinger’s CATalytic Pocket. First revealed by the computational-biochemical-evolutionary method, the pocket dynamic has since been independently confirmed by structural analyses of A3s.

In a study in 2017, to examine whether Hs-AID’s unique biochemical properties (i.e., low catalytic rate and high affinity for its substrate) were conserved across vertebrates, we compared the enzymatic activity of Hs-AID to that of sea lamprey, nurse shark, and coelacanth. These species were chosen to represent key points of evolution, lamprey being a jawless vertebrate, shark being the first jawed vertebrate with the classical Ig system, and coelacanth being the “fossil fish” lobe fined fish thought to be the closest fish ancestor of tetrapods (21). We found that despite the biochemical variability amongst these enzymes in substrate sequence preference (WRC *vs*. non-WRC motifs) and optimal temperatures, the key defining enzymatic characteristics of AID (lethargic catalytic rate and high nM range affinity for ssDNA binding) were maintained (205). This finding suggests that these unique biochemical regulatory features of low catalytic rate and high ssDNA binding affinity in AID are evolutionary conserved and thus important for its function, for instance the balance between making SHM and CSR mutations while protecting the genome from rampant promiscuous mutagenesis. Furthermore, using computational modelling, we showed that all of the above-mentioned AIDs are predicted to exhibit the Schrödinger’s CATalytic Pocket phenomenon, revealing the importance of this intrinsic structural regulatory mechanism for AID activity throughout the vertebrate class (205). Importantly, this was also the first study to show that two species, key in the evolution of adaptive immunity in its classical mammalian form, the shark and the coelacanth, do indeed have a functional AID enzyme.

In a more recent study, colleagues and we turned our focus to the extant agnathan the sea lamprey, in which thus far two AID-like cytidine deaminases (CDA1 and CDA2) have been found (20). Genetic analyses revealed that CDA1 and CDA2 were found in both the sea and freshwater lampreys, along with, unexpectedly, multiple CDA1-like genes that could be divided into two distinct groups (CDA1L1_1, _2, _3, _4 and CDA1L2_1, _2). Genomic DNA from other individuals were searched for homologs of these new CDA1-like genes, which were found, along with splice variants of CDA1L1_1 and CDA1L1_3. When their amino acid sequences were compared with those of other AID orthologs, these novel CDA-like proteins were found to contain the conserved deaminase core catalytic motif (HxEx_n_PCxxC), suggesting they could be active cytidine deaminase. *In silico* modeling of each CDA ortholog also demonstrated the high likelihood of catalytic cytidine deaminase activity, as each protein formed a putative cytidine deaminase catalytic site, and catalytic activity was demonstrated by expressing these enzymes in 293T cells and assaying the extracts for cytidine deaminase activity. The enzymes exhibited cold adaptation, with optimal temperatures being between 14-22°C, and most had an acid pH-adapted activity profile, reminiscent of the human A3 branch enzymes (A3A, A3B, A3G, A3F) rather than human AID, and commensurate with structural modeling showing that these proteins have a lower surface charge than human AID. These results showed for the first time that lamprey has more than just one version of a CDA1 enzyme, and remarkably, that these are variably expressed in individuals of the same species, a novel biological phenomenon the mechanism and importance of which is yet to be discovered.

## Discussion

**Figure 2:**
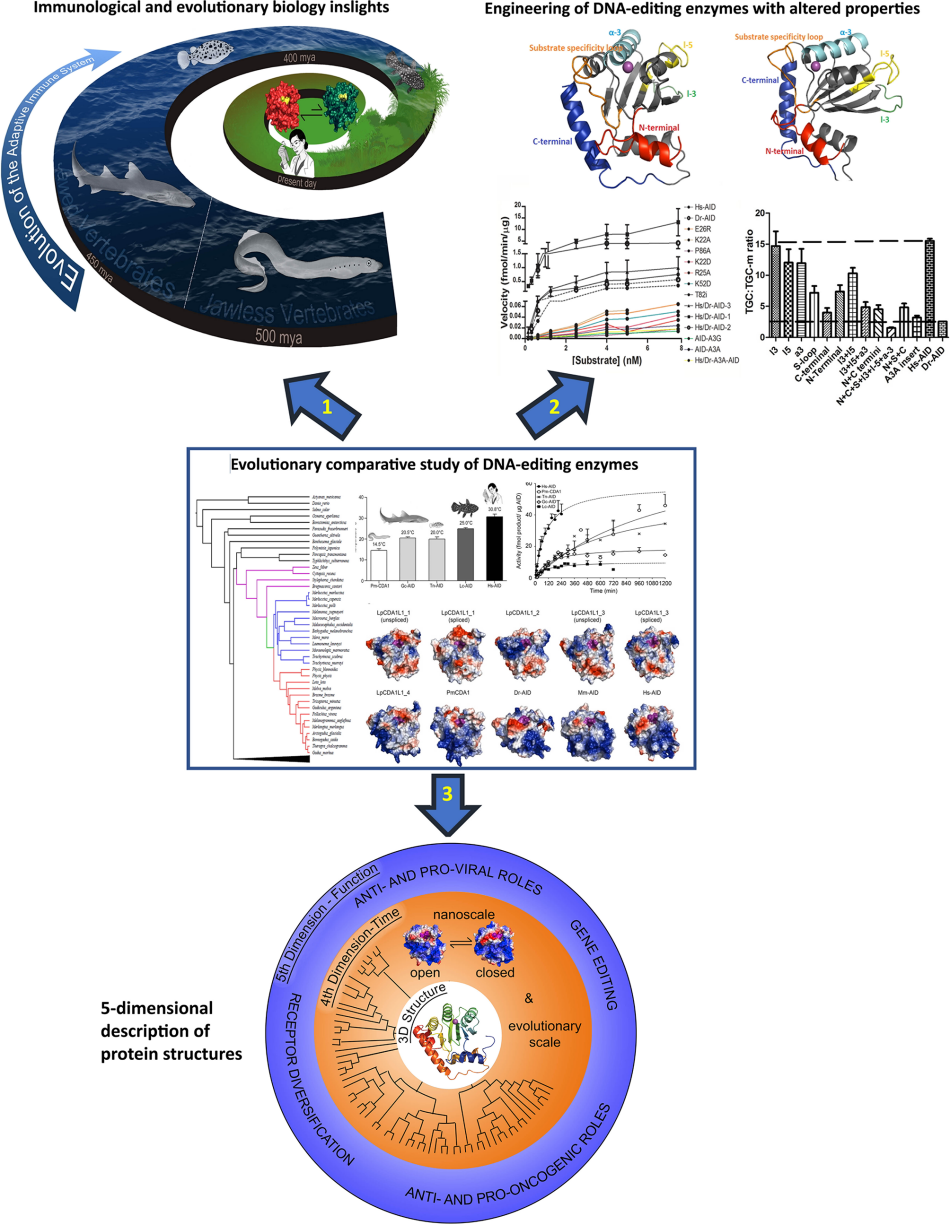
The concept and three main applications of the evolutionary-biochemical-computational approach to studying DNA-editing enzymes. The evolutionary comparative approach is shown in the middle with 3 arrows each pointing to an area wherein this approach can make significant impact. The evolutionary comparative approach shown in the middle panel consists of comparing biochemical properties (Michaelis-Menten kinetics, substrate binding kinetics, optimal temperature, optimal pH, substrate sequence or shape specificity, etc.) of the enzymes using enzyme assays and considering insights in the context of their 3D solved structures or computational predicted models as shown in this figure. Due to vast biochemical diversity observed amongst various AID orthologs, examining the biochemical properties of divergent AID orthologs has shed light on many structure:function aspects of AID/APOBEC enzymes. Arrow 1: the evolutionary comparative study of DNA-editing enzymes can provide insights into the evolution of the immune system, for instance on whether the immune systems use active deaminases and how/if they have gene sequences or other immune genes that have co-evolved with their deaminases. Arrow 2: using different orthologs allows for generation of libraries of mutants and chimeric enzymes which can have diverse biochemical properties such as DNA/RNA-targeting profiles and sequence specificities, and these can be used for applications such as base editing. Arrow 3: the most important highlight of the evolutionary-biochemical-computational approach is the birth of the concept of 5-dimentional (5D) structural description, proposed in this article. The 5D description integrates the classical 3D structure of a protein with dynamic changes in time (4^th^ dimension) and the relevance of these to function (5^th^ dimension). The middle panel contains reproduced figures from previous publications. The thermosensitivity and enzyme velocity plots are from our previous work Quinlan EM et al. (21). Biochemical regulatory features of activation-induced cytidine deaminase remain conserved from lampreys to humans. Mol Cell Biol 37:e00077-17. https://doi.org/10.1128/MCB.00077-17. Copyright ^©^ 2017 American Society for Microbiology. The computational models are adapted from our previous work Holland et al. (20). Expansions, diversification, and interindividual copy number variations of AID/APOBEC family cytidine deaminase genes in lampreys. 2018 Apr 3;115(14):E3211-E3220. doi: 10.1073/pnas.1720871115 Copyright (2018) National Academy of Sciences.

In the above sections, we reviewed the insights relevant to structural biology, immunology, and cancer research that have been brought forth by comparative studies of AID from non-human/mouse species. This section highlights the future potential of comparative evolutionary studies for impacting emerging approaches in structural biology, base-editing, and protein engineering. The concept of how evolutionary studies illuminate each of these three arenas is illustrated in **Figure 2**.

**Figure 3:**
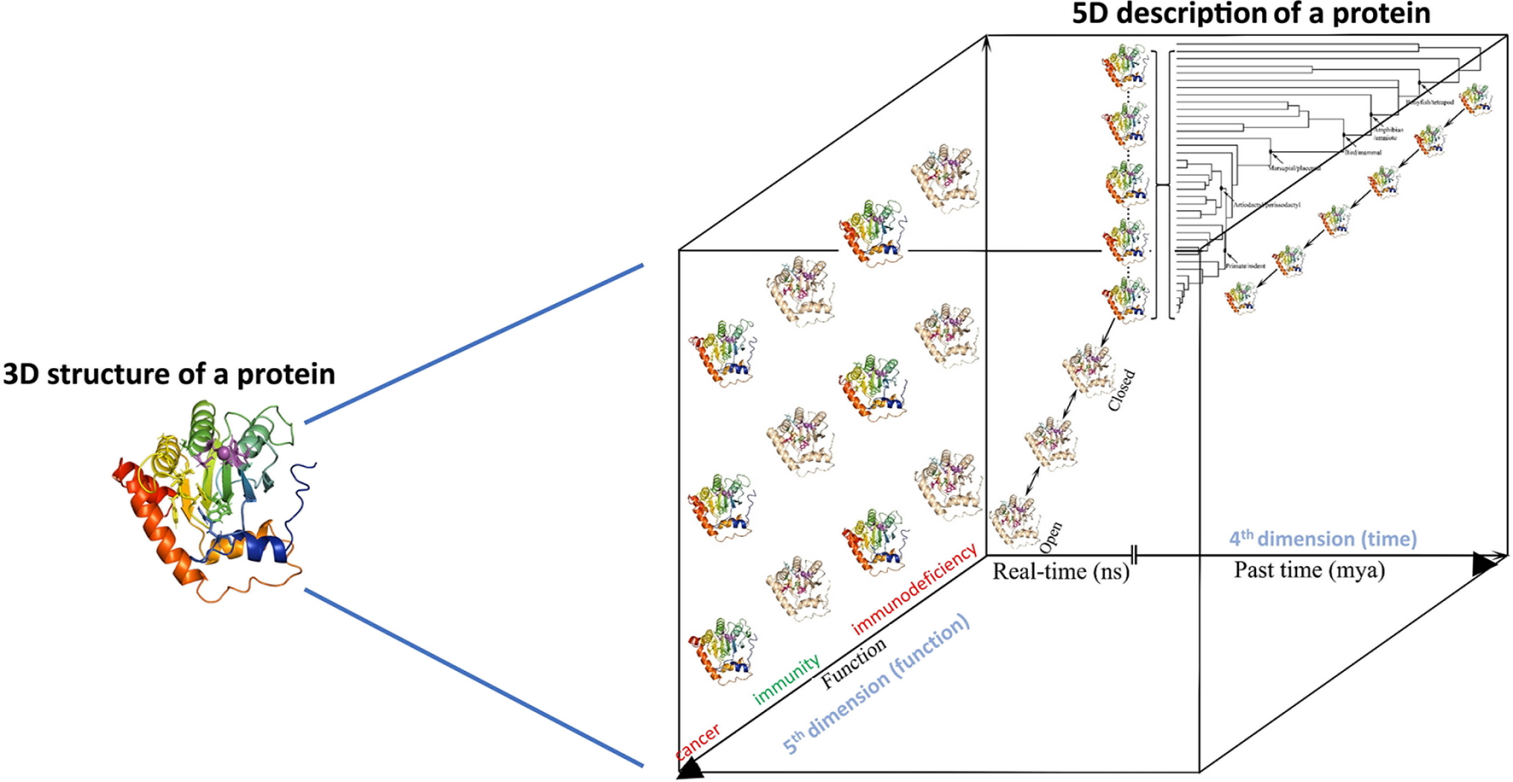
5-dimensional description of biological molecules. In the 5D structure description proposed here, the information from the traditional 3D structure is combined with the structure dynamics in time (4^th^ dimension = time, in real time measured in fractions of a second, and evolutionary time measured in millions of years) and integrated with how these real-time and evolutionary dynamic structural changes impact the biological function of the protein (5^th^ dimension = biological function as dependent on 3D and 4D descriptions of a protein’s structure).

First, with respect to structural biology (**Figure 2** arrow 3, bottom panel, and **Figure 3**), the significance of this computational-biochemical-evolutionary approach for AID is evident by its track record of providing the first 3D map of AID structure and revealing the concept of Schrödinger’s CATalytic Pocket in the AID/APOBEC family, both of which have subsequently been confirmed by independent studies employing the traditional structure solution methods of crystallography and NMR. Thus, in the case of AID, not only did the evolutionary-biochemical-computational approach for solving its structure prove to be quicker, it was also the only approach able to deal with AID in an unaltered native state, as the only way to crystalize AID has been to alter it, with the most near-native crystal structure still containing 20 aa truncation and multiple surface mutations that change the charge of native AID drastically (from +11 to +3) (52, 129). Furthermore, the evolutionary-biochemical-computational method also revealed additional time/space dimensions of the structure that are not normally probed through the traditional methodologies (53). For this reason, we termed this type of computational-biochemical-evolutionary structure a five-dimensional (5D) description of a 3D structure. In the 5D structure of a protein, as opposed to the classical protein structure which has always been viewed as a 3D shape, the structure’s dynamics are further explored through time (4^th^ dimension) dimensions of ‘*tempus*’ and ‘*aevum*’. The ‘*tempus*’ analysis is the studying of a protein structure in a real time manner where one can examine/predict the ‘protein breathing’ on the time scale of fractions of a second, while the ‘*aevum*’ sub-dimension is one wherein dynamic change is compared throughout ortholog evolution from both closely- and remotely-related species, on the time scale of hundreds of millions of years. The 5^th^ dimension, which is “function”, then explores the understanding of how these dynamic 3D and 4D structures relate to the biological function of the protein, including functions in human health/disease. **Figure 3** illustrates the concept of how a 5D structure description contains orders of magnitude more information than the conventional 3D picture.

Others and we have shown that AID orthologs exhibit a vast diversity in many of their biochemical properties such as catalytic rate, optimal temperature, optimal pH, and substrate sequence specificity. Indeed, the catalytic rate varies over 3 orders of magnitude, temperature optima vary from very cold to human body temperature, and pH optima vary over a range of nearly 2 units. Firstly, this is indeed a remarkable range of variation for evolutionary closely related versions of the same enzyme, given that a large portion of the enzyme’s primary sequence and its overall 3D structural architecture are conserved. Secondly, each of these biochemical characteristics is an indicator of a specific structural aspect of a protein. For instance, variations in catalytic rate can be due to differences in substrate binding or differential dynamics of the catalytic pocket as dictated by breathing loops that compose the catalytic pocket. Variations in optimal pH are largely owing to the surface charge of the protein, which in AID can vary from only slight positive in some bony fish (e.g., +3 in *Salmo Salar*) to extremely positive (e.g., +11 in human and mouse). Substrate specificity differences are mediated by a well-defined substrate specificity loop which is one of the more variable structural regions among the AID/APOBEC family members, causing different surface binding pockets next to the catalytic pocket that underlie differential preference for the -2 and -1 base positions next to the target dC that is positioned in the catalytic pocket. For temperature sensitivity, proteins may increase their thermoresistance using several strategies. In the first mechanism, the enthalpy change (ΔH_s_) measured at the temperature of maximum stability (T_s_) becomes more negative, causing ΔG for all temperatures to decrease. This strategy can be seen as a stability curve to be shifted downward. The second strategy is to increase (less negative) the change in the heat capacity upon folding (ΔC_p_) which causes T_m_ to increase. In this case, the stability curve would broaden. The last approach is to increase T_s_ which shifts the curve to the right. Proteins may apply one, two, or all of these strategies to improve their thermal resistance, and this is dictated by differences in the secondary structures employed in various parts of the protein and/or overall flexibility of the structure. Thus, not only is each of an enzyme’s biochemical properties reflective of a structural trait in terms of the 3D folding of the structure, but the relationship between properties (e.g., between catalytic rate and optimal temperature) itself can also provide finer level information into the subtle differences of 3D folding of the enzyme’s protein structure.

Modelling of proteins with no known related structure is a long-standing challenge in the field of structural biology where the recent breakthrough of AlpahFold has gained considerable attention (206). The AlphaFold algorithm, a learned-based method in contrast to knowledge- and physics-based ones, uses co-evolution methods and deep convolutional neural networks. Remarkably, combining the deep-learning methods such as AlphaFold with molecular dynamics stimulations has improved the accuracy of protein structural prediction even further (207). However, to achieve an accurate result using learned-based methods, access to a large dataset (e.g., multiple sequence alignment [MSA] of 10^5^ to 10^6^ sequences) of evolutionarily diverse sequences is necessary (208). Co-evolution-derived contact methods are based on the idea that the residues in close contact (< 8 Å considering the Cα) in the 3D structure, which define the local secondary structural features, co-evolve while the residues with medium- and long-range contact specify the overall 3D structure of a protein. In fact, the evolutionarily conserved dynamical/functional domains (termed evolutionary domains [ED]) have been predicted by coevolutionary coupling analysis of co-evolving residues (209). The contact map of the protein can be retrieved either through the evolutionary coupling analysis (ECA) or supervised machine learning (SML). ECA relies on a high-quality large MSA (with at least 64 times the square root of the length of the target protein) while SML methods are capable of retrieving the contact map even in the case of smaller MSA by combining the sequence-dependent and independent information (210). Therefore, the approach of studying a family of proteins from many orthologs that cover a large range of biochemical properties, coupled with artificial intelligence (AI) learning, will pave the road for even more refinement of such AI-based computational approaches to protein folding, and especially so in the field of enzymology. Given that this methodology may be nearing the accuracy of experimental structure determination, as announced recently, and the applicability of enzyme (e.g., virus polymerases) structure prediction and engineering for treatment of emerging pathogens, the evolutionary comparative study of enzymes can make a critical contribution in this domain.

From a basic evolutionary immunology perspective (**Figure 2**, arrow 1, top left), the comparative enzymology approach also has brought forth meaningful insights and points for further research. For instance, the discovery that AID’s catalytic pocket has evolved in one fish species to be significant more active, or capable of carrying out genome demethylation, speaks to issues of DNA-repair and genome demethylation that provide hints that in some instances in evolution, AID may indeed have had a significantly higher weight of non-immune based physiological functions as compared to the case in mammals where it plays a strictly immune role. Though other roles such as epigenetic remodelling have been proposed for human and mouse AID, the fact that AID-deficient mice appear only to suffer from Hyper IgM and no other perturbations suggest that any non-immune functions of AID in mammals are either marginal or highly redundant. This in turn suggests that perhaps AID initially emerged for other functions in the fish and was later co-opted by the immune system, a familiar pattern, that has already been shown for other DNA-damaging enzymes used by the immune system, namely the RAG recombinases. Demonstrating that AID is an active deaminase in species like sharks and coelacanth, which are key fishes in the evolution of vertebrates, also shed light on AID’s role in earlier-evolved immune systems. Lastly, the unexpected and novel expansion and inter-individual copy number variation of the AID-like CDA1 enzymes in the lamprey speaks to the intriguing possibility that somehow the enzymes themselves may be the subject of an as-yet-undiscovered type of genetic diversification or environmental response.

From the perspective of protein biotechnological advancements in protein engineering (**Figure 2**, arrow 2, top right), the comparative evolutionary enzymology method is also of value for emerging biotechnological applications, such as in the emerging field of base-editing. DNA base editing is a new genome editing tool, introduced in 2016, based on the clustered regularly interspaced short palindromic repeats (CRISPR) associated (Cas) system of bacterial adaptive immunity, where a point mutation is precisely introduced into the genomic DNA (211–214). This tool is comprised of a guide RNA, a catalytically impaired Cas nuclease coupled to a ssDNA mutating enzyme. There are two different classes of ssDNA base editors, the cytidine base editors (CBEs) and adenine base editors (ABEs), where different deaminases are used as the ssDNA mutating enzyme (215). CBEs accomplish the conversion of C:G to T:A using cytidine deaminases (i.e., AID/APOBEC family members) while ABEs perform the reverse mutation using adenine deaminases (e.g., TadA). The specificity of the CBE complexes is defined by the protospacer adjacent motif (PAM) which is recognized by the Cas enzyme, the activity-window which is defined by the target sequence incorporated into the single-guide RNA (sg-RNA), and the substrate specificity of the ssDNA mutating enzyme. Since the sequence content of the target dC is defined by the target genomic regions, diversifying the substrate specificity of the ssDNA editing enzymes are of a great interest. To accomplish this goal, different members of the AID/APOBEC family, such as AID, APOBEC1, A3A, 3B, 3C, 3D, 3F, 3G, 3H, and CDA1 from human, rat, and sea lamprey, and their variants have been tethered to Cas. Deamination of methylated dC was also accomplished by using A3A variants as the ssDNA editing enzyme (216). Given the observed diversity in the biochemical properties of AID orthologs, using AID from different species, especially bony fish, would assist in expanding the specificity of the CBEs arsenal. A recent study acts as evidence for this; the study did a screen of 153 *in vitro*-evolved cytidine deaminases (APOBECs, AIDs, CDAs, etc.), led to ones that exhibited the lowest unguided off-target DNA and cellular RNA deamination events along with the highest on-target deamination events. Using this screening approach to choosing a ssDNA editing enzyme, it became possible to reduce the unguided off-target DNA deamination events by 45-fold and transcriptome-wide deamination events by 12- to 69-fold, all while maintaining a similar DNA on-target editing frequency (212). Others and we who have been studying cytidine deaminase structure:function and evolution have also generated libraries of chimeric and mutant enzymes, bearing different motifs exchanged between orthologs in order to pinpoint enzyme functionality to structural parts (**Figure 2**, arrow 2, top right). In so doing, these libraries often contain engineered enzymes with variable targeting and substrate specificity profiles that could also prove as useful tools in the field of base-editing.

In conclusion, molecules involved in human health and disease are typically studied in only a handful of well-characterized model species. Here, using the example AID, a DNA-editing enzyme involved in immunity and cancer, we have reviewed how the few studies that have examined this molecule in evolutionarily distant species have brought forth important and unexpected insights in structural biology, immunology, and cancer research. For other DNA-damaging enzymes involved in immunity and cancer, such as RAGs, the case is parallel, with less than a handful of hundreds of studies probing non-mouse/human species; however, the studies that have ventured into the evolutionary past have brought forth intriguing ideas that have changed our understanding of RAG function and evolution (217–221). This, taken together with the fact that by far the greatest window of evolutionary diversity in these DNA-editing proteins, and indeed in all proteins, lies in earlier-evolved species that have remained unstudied, would make it reasonable to conclude that much fundamental and applicable biological insights can be uncovered by large-scale evolutionary studies. The case of understudied orthologs discussed here (**Figure 1B**) is made even more glaring considering that unlike the field of evolutionary immunology which is a recognized subfield of immunology with its own research groups, journals and scientific meetings, other disciplines such as DNA repair, cancer research, neurodegenerative diseases, and many others do not have a formal evolutionary sub-discipline. We have also discussed how, in addition to generating novel fundamental knowledge on biology, the evolutionary comparative approach for studying protein structure:function is a valuable tool to complement the emerging AI-guided protein folding methodologies as well as protein engineering in the field of base-editing and beyond.

## Author Contributions

All authors contributed to the preparation of the text and figures. All authors contributed to the article and approved the submitted version.

## Funding

Natural Sciences and Engineering Research Council of Canada (NSERC), Grant/Award Number: 2015-047960.

## Conflict of Interest

The authors declare that the research was conducted in the absence of any commercial or financial relationships that could be construed as a potential conflict of interest.
